# Antibodies against specific extractable nuclear antigens (ENAs) as diagnostic and prognostic tools and inducers of a profibrotic phenotype in cultured human skin fibroblasts: are they functional?

**DOI:** 10.1186/s13075-019-1931-x

**Published:** 2019-06-24

**Authors:** Claudio Corallo, Sara Cheleschi, Maurizio Cutolo, Stefano Soldano, Antonella Fioravanti, Nila Volpi, Daniela Franci, Ranuccio Nuti, Nicola Giordano

**Affiliations:** 1 Scleroderma Unit, Department of Medicine, Surgery and Neurosciences, University of Siena, Siena, Italy; 2 Rheumatology Unit, Department of Medicine, Surgery and Neurosciences, University of Siena, Siena, Italy; 3 Research Laboratory and Academic Division of Clinical Rheumatology, Department of Internal Medicine, University of Genoa, Genoa, Italy

**Keywords:** Systemic sclerosis, Fibrosis, Autoantibodies, Fibroblasts, Centromeric protein B, Topoisomerase I

## Abstract

**Background:**

The importance of systemic sclerosis (SSc) autoantibodies for diagnosis has become recognized by their incorporation into the 2013 ACR/EULAR classification criteria. Clear prognostic and phenotypic associations with cutaneous subtype and internal organ involvement have been also described. However, little is known about the potential of autoantibodies to exert a direct pathogenic role in SSc. The aim of the study is to assess the pathogenic capacity of anti-DNA-topoisomerase I (anti-Topo-I) and anti-centromeric protein B (anti-Cenp-B) autoantibodies to induce pro-fibrotic markers in dermal fibroblasts.

**Methods:**

Dermal fibroblasts were isolated from unaffected and affected skin samples of (*n* = 10) limited cutaneous SSc (LcSSc) patients, from affected skin samples of diffuse cutaneous (DcSSc) patients (*n* = 10) and from healthy subjects (*n* = 20). Fibroblasts were stimulated with anti-Topo-I, anti-Cenp-B IgGs, and control IgGs in ratios 1:100 and 1:200 for 24 h. Cells were also incubated with 10% SSc anti-Topo-I^+^ and anti-Cenp-B^+^ whole serum and with 10% control serum for 24 h. Viability was assessed by MTT test, while apoptosis was assessed by flow cytometry. Activation of pro-fibrotic genes ACTA2, COL1A1, and TAGLN was evaluated by quantitative real-time PCR (qPCR), while the respective protein levels alpha-smooth-muscle actin (α-SMA), type-I-collagen (Col-I), and transgelin (SM22) were assessed by immunocytochemistry (ICC).

**Results:**

MTT showed that anti-Cenp-B/anti-Topo-I IgGs and anti-Cenp-B^+^/anti-Topo-I^+^ sera reduced viability (in a dilution-dependent manner for IgGs) for all the fibroblast populations. Apoptosis is induced in unaffected LcSSc and control fibroblasts, while affected LcSSc/DcSSc fibroblasts showed apoptosis resistance. Basal mRNA (ACTA2, COL1A1, and TAGLN) and protein (α-SMA, Col-1, and SM22) levels were higher in affected LcSSc/DcSSc fibroblasts compared to LcSSc unaffected and to control ones. Stimulation with anti-Cenp-B/anti-Topo-I IgGs and with anti-Cenp-B^+^/anti-Topo-I^+^ sera showed a better induction in unaffected LcSSc and control fibroblasts. However, a statistically significant increase of all pro-fibrotic markers is reported also in affected LcSSc/DcSSc fibroblasts upon stimulation with both IgGs and sera.

**Conclusions:**

This study suggests a pathogenic role of SSc-specific autoantibodies to directly induce pro-fibrotic activation in human dermal fibroblasts. Therefore, besides the diagnostic and prognostic use of those autoantibodies, these data might further justify the importance of immunosuppressive drugs in the early stages of the autoimmune disease, including SSc.

**Electronic supplementary material:**

The online version of this article (10.1186/s13075-019-1931-x) contains supplementary material, which is available to authorized users.

## Background

Systemic sclerosis (scleroderma, SSc) is a rare and heterogeneous autoimmune disease characterized by progressive fibrosis of the skin and internal organs such as lungs, heart, kidneys, and gastrointestinal tract, coupled to widespread vascular alterations [1]. The main abnormalities of SSc are related to the connective tissue, in which the excessive production of collagen and other extracellular matrix components are responsible for a progressive and, so far irreversible, fibrosis [2]. The clinical phenotype of SSc varies between two main distinct subsets according to the extent of the skin involvement [3]: limited cutaneous systemic sclerosis (LcSSc) in which skin thickening is mainly restricted to the face, fingers, and forearms [4] and diffuse cutaneous systemic sclerosis (DcSSc) in which skin lesions are observed on the trunk and over the elbow and/or knee [5]. It is well known that SSc has an autoimmune etiology: anti-nuclear antibodies (ANAs) are detected in more than 95% of patients [6] and the presence of several potentially pathogenic auto-antibodies targeting various extractable nuclear antigens (ENAs) or other auto-antigens is also reported [7]. ANAs in SSc are divided into two categories: SSc-specific ANAs and SSc-associated ANAs [8]. SSc-specific ANAs are detected in SSc patients and rarely found in other connective tissue diseases or in healthy subjects [9]. They include anti-centromere (ACAs) and anti-DNA topoisomerase I (Topo-I) antibodies mainly, but also anti-RNA polymerase III (RNAP), anti-U3 ribonucleoprotein (RNP), anti-Th/To, anti-U11/U12 RNP, anti-eukaryotic initiation factor 2B (eIF2B), anti-U1 RNP, anti-PM-Scl, anti-Ku, and anti-RuvBL1/2 antibodies for the minor component. On the other hand, SSc-associated ANAs are not specific to SSc but they can occasionally coexist with other connective tissue disease-related antibodies [10, 11]. The two main subsets of SSc (LcSSc and DcSSc) do not reflect only a clinical classification [3], but they are usually associated with a precise autoimmune pattern: in fact, ACAs and in particular anti-centromere B (anti-Cenp-B) antibodies are predominantly associated with LcSSc, while anti-Topo-I with DcSSc [12]. In fact, while the other SSc-specific or SSc-associated antibodies can be found in both LcSSc and DcSSc, it is less frequent to find LcSSc patients with anti-Topo-I antibodies and DcSSc patients with anti-Cenp-B antibodies [13]. The utility of SSc-specific antibodies for both diagnostic and prognostic purposes has been fully elucidated [14]. In fact, the ACR/EULAR 2013 classification criteria now include the presence of ACAs, anti-Topo-I, and anti-RNAP-III antibodies as one of the items to overcome the disadvantages of the 1980 ACR preliminary classification criteria [15, 16]. Regarding the prognostic utility, the faster progression of the disease in SSc-specific antibody-positive patients versus SSc-specific antibody-negative ones has been demonstrated [17]. ANA-negative SSc patients (5%) represent one unique subgroup mainly characterized by male subjects with less vasculopathy, such as digital ulcers and pulmonary arterial hypertension (PAH), and with lower gastrointestinal involvement [18]. Whether ANA-negative SSc patients have other non-nuclear circulating antibodies has not been currently elucidated [19]. Taken into account the abovementioned diagnostic and prognostic utilities of SSc-specific antibodies, the goal of the present study is to investigate whether those antibodies could have a direct pathogenic effect on in vitro cultured human fibroblasts.

## Materials and methods

### Patients and cell cultures

Ten patients with LcSSc and ten patients with DcSSc who fulfilled the 2013 ACR/EULAR classification criteria for SSc [15, 16], and ten age- and sex-matched voluntary healthy subjects were recruited from the Internal Medicine and Rheumatology Units of the University Hospital of Siena in accordance with the Declaration of Helsinki, after obtaining signed informed consent and following the local Ethical Board Committee approval. Clinical and demographical characteristics of SSc patients are reported in Table 1. Blood was collected from SSc patients and from healthy subjects, and serum samples were checked for the presence of autoantibodies according to normal diagnostic procedures. Skin biopsies were performed using a 3-mm punch on the affected mid-forearm of patients with LcSSc/DcSSc. Unaffected areas of skin from the same LcSSc patients and control skin (site-matched) from gender- and age-matched healthy subjects were also evaluated. The LcSSc patients’ unaffected skin was defined by clinical palpation and graded as zero on the modified Rodnan skin score [20]. Fibroblasts were isolated from skin specimens by enzymatic digestion. Briefly, explants were de-epidermized using a dispase solution (dispase activity 14 U/mL) (Sigma-Aldrich, St. Louis, MO, USA) for 2 h at 37 °C and then were dissolved into a type IV collagenase solution (2.4 U/mL) (Sigma-Aldrich) for 3 h.

**Table 1:** Demographic and clinical characteristics of patients at the time of biopsy collection

Patients	Subset	Age (years)	Gender (M/F)	Disease duration (years)	Autoantibody	mRSS	Organ involvement	Therapy
1	LcSSc	57	F	12	Anti-Cenp-B	17	Lung (ILD), digital ulcers	ERAs, prednisone
2	LcSSc	62	F	5	Anti-Cenp-B	4	Esophagus	Ca2+ antagonists, PPIs
3	LcSSc	70	M	9	Anti-Cenp-B	14	Lung (ILD), esophagus	ERAs, PPIs, MMF
4	LcSSc	67	F	10	Anti-Cenp-B	5	Esophagus	Ca_2+_ antagonists, PPIs
5	LcSSc	48	F	8	Anti-Cenp-B	14	Lung (ILD)	ERAs, Prednisone, MMF
6	LcSSc	55	F	11	Anti-Cenp-B	7	Lung (ILD)	ERAs, Prednisone
7	LcSSc	58	F	13	Anti-Cenp-B	17	Lung (ILD), esophagus	ERAs, PPIs, MMF
8	LcSSc	44	F	2	Anti-Cenp-B	7	Lung (PAH), esophagus	Ca_2+_ antagonists, PPIs, prostanoids
9	LcSSc	66	F	7	Anti-Cenp-B	14	Digital ulcers esophagus	ERAs, PPIs
10	LcSSc	60	F	9	Anti-Cenp-B	5	Esophagus	Ca_2+_ antagonists, PPIs
11	DcSSc	51	F	12	Anti-Topo-I	7	Lung (PAH), digital ulcers	Ca_2+_ antagonists, ERAs, prostanoids
12	DcSSc	72	M	11	Anti-Topo-I	17	Lung (PAH), digital ulcers	ERAs, prostanoids, MMF
13	DcSSc	64	F	7	Anti-Topo-I	17	Lung (PAH), digital ulcers	ERAs, prostanoids, MMF
14	DcSSc	63	M	13	Anti-Topo-I	12	Lung (ILD)	ERAs, prednisone, MMF
15	DcSSc	50	F	6	Anti-Topo-I	9	Lung (ILD), esophagus	Ca_2+_ antagonists, PPIs
16	DcSSc	68	F	15	Anti-Topo-I	14	Lung (ILD)	ERAs, prednisone, MMF
17	DcSSc	59	F	14	Anti-Topo-I	17	Esophagus	Ca_2+_ antagonists, PPIs
18	DcSSc	69	M	7	Anti-Topo-I	17	Lung (PAH)	ERAs, prostanoids
19	DcSSc	49	F	9	Anti-Topo-I	14	Lung (PAH), esophagus	ERAs, prostanoids, PPIs
20	DcSSc	61	F	11	Anti-Topo-I	17	Lung (ILD), esophagus	Ca_2+_ antagonists, PPIs, MMF

*Abbreviations*: *Cenp-B* centromeric protein B, *ERAs* endothelin receptor antagonists, *ILD* interstitial lung disease, *MMF* mycophenolate mofetil, *mRSS* modified Rodnan skin score, *PPIs* proton pump inhibitors, *PAH* pulmonary arterial hypertension, *Topo-I* topoisomerase I

The obtained cell suspension was filtered twice using 70-μm nylon meshes, washed, and centrifuged for 5 min at 700×*g*. The viability was assessed by Trypan Blue (Sigma-Aldrich) test identifying 90 to 95% cell survival. Fibroblasts were recovered, seeded into 10-cm diameter tissue culture plates, and were expanded twice and cultured in a monolayer incubator with 5% CO_2_ and 90% humidified atmosphere at 37 °C until confluence. Cells were grown in Dulbecco’s modified Eagle medium (DMEM) (Euroclone, Milan, Italy), containing 10% fetal bovine serum (FBS) (Euroclone), with 200 U/mL penicillin and 200 μg/mL streptomycin (Sigma-Aldrich) and 2 mM glutamine (Sigma-Aldrich). The medium was changed every 3–4 days. The fibroblast morphology was examined daily with an inverted microscope (Olympus IMT-2, Tokyo, Japan) to guarantee their phenotypic stability preserved. For each single experiment, a cell culture from a unique donor was used.

### Fibroblast treatment

Fibroblasts at the third passage were employed for the experiments. Twelve hours before the experiments, cells were harvested in a serum-free medium and cultured in 75 cm^2^ flasks (Euroclone, Milan, Italy).

Human polyclonal anti-centromere B (anti-Cenp-B) and anti-DNA topoisomerase I (anti-Topo-I) (Abcam, Cambridge, UK) were first dissolved in phosphate-buffered saline (PBS) (Euroclone), according to the manufacturer’s instructions, and then they were diluted in the culture medium immediately before the treatment to reach the final dilution required (1:100 and 1:200). The cells were treated with the conditioned media containing the selected dilutions of anti-Cenp-B and anti-Topo I and 10% SSc anti-Topo-I^+^, anti-Cenp-B^+^ serum for 24 h. The final concentrations were chosen based on the best results obtained in terms of viability (data not shown). To evaluate the effect of antibodies directed against nuclear proteins which are not involved in SSc pathophysiology, a human anti-histone H3 antibody (Abcam) was also tested in parallel in control fibroblasts only (Additional file 1).

After the treatment, the media were removed, centrifuged, and stored at − 80 °C; the fibroblasts were immediately processed to carry out cell viability assay, flow cytometry analysis, quantitative real-time PCR, and immunofluorescence analysis.

### MTT assay

The viability of the cells was evaluated immediately after the treatment by MTT assay. Fibroblasts from LcSSc/DcSSc patients and healthy subjects were seeded in 12-well plates (8 × 10^4^ cells/well) for 24 h in DMEM with 10% FBS. Then, the medium was removed, and the cells were cultured in DMEM with 0.5% FBS usually used during the treatment procedure. After that, the cells were incubated for 3 h at 37 °C in a culture medium containing 10% of 5 mg/mL MTT (3-[4,4-dimethylthiazol-2-yl]-2,5-diphenyl-tetrazoliumbromide) (Sigma-Aldrich). After the period of incubation, the medium was removed and 0.2 mL of dimethyl sulfoxide (DMSO) (Rottapharm Biotech, Monza, Italy) was added to each well to solubilize the formazan crystals. The absorbance was measured at 570 nm in a microplate reader (BioTek Instruments, Inc., Winooski, VT, USA). A control well without cells was employed for blank measurement. The percentage of survival cells was evaluated as follows: % of survival cells = (absorbance of considered sample) / (absorbance of control) × 100. The experiments were performed on sub-confluent cell cultures in order to prevent contact inhibition which can condition the results. Data were normalized and reported as optical density (OD) units per 10^4^ adherent cells.

### Detection of apoptosis

The evaluation of apoptotic cells was developed by using Annexin V-FITC and propidium iodide (PI) (Thermo Fisher Scientific, Milan, Italy). Fibroblasts from LcSSc/DcSSc patients and healthy subjects were seeded in 12-well plates (8 × 10^4^ cells/well) for 24 h in DMEM with 10% FBS. Then, the medium was removed, and the cells were cultured in DMEM with 0.5% FBS usually used during the treatment procedure described before. After that, the fibroblasts were washed and harvested by using trypsin, collected into cytometry tubes, and centrifuged at 1500 rpm for 10 min. The supernatant was replaced, and the pellet was resuspended in 100 μL of 1× Annexin-binding buffer, 5 μL of Alexa Fluor 488 annexin-V conjugated to fluorescein (green fluorescence), and 1 μL of 100 μg/mL PI working solution. Cells were incubated at room temperature for 15 min in the dark. Then, 600 μL of 1× Annexin-binding buffer was added before the analysis at flow cytometer. A total of 10,000 events (1 × 10^4^ cells per assay) were measured by the instrument. The obtained results were analyzed with Cell Quest software (Version 4.0, Becton Dickinson, San Jose, CA, USA). The evaluation of apoptosis was carried out considering staining cells simultaneously with Alexa Fluor 488 annexin-V and PI; this allowed to discriminate intact cells (annexin-V and PI-negative), early apoptosis (annexin-V-positive and PI-negative), and late apoptosis (annexin-V and PI positives) [21]. The results were normalized per 10^4^ cells and expressed as a ratio of positive cells to each dye (total apoptosis), and the data were represented as the mean of three independent experiments (mean ± standard deviation (SD)). To determine the impact of apoptosis on fibroblast pro-fibrotic activation, inhibitor of apoptosis (IAP) AZD 5582 dihydrochloride (Sigma-Aldrich) compound was added to control fibroblasts 2 h before the stimulation with anti-Cenp-B (1:100) and anti-Topo-I (1:100) IgGs. The final concentration chosen is 50 nM after appropriate dose finding (Additional file 1).

### RNA isolation and quantitative real-time PCR

Fibroblasts from LcSSc/DcSSc patients and healthy subjects were seeded in 6-well dishes at a starting density of 6 × 10^6^ cells/well for 24 h in DMEM with 10% FBS. Then, the medium was removed, and the cells were cultured in DMEM with 0.5% FBS usually used during the treatment procedure.

Total RNA was extracted using TriPure Isolation Reagent (Euroclone) according to the manufacturer’s instructions and was stored at − 80 °C. The concentration, purity, and integrity of RNA were evaluated by measuring the OD at 260 nm and the 260/280 and 260/230 ratios by Nanodrop-1000 (Celbio, Milan, Italy). The quality of RNA was verified by electrophoresis on agarose gel (Flash Gel System, Lonza, Rockland, ME, USA). Reverse transcription for target genes was carried by QuantiTect Reverse Transcription Kit (Qiagen, Hilden, Germany), according to the manufacturer’s instructions.

Then, target genes were examined by real-time PCR by using QuantiFast SYBR Green PCR (Qiagen) kit. A list of the used primers is reported in Table 2. All qPCR reactions were achieved in glass capillaries by a LightCycler 1.0 (Roche Molecular Biochemicals, Mannheim, Germany) with LightCycler Software Version 3.5. The reaction procedure for target gene amplification was performed at 5 in at 95 °C, 40 cycles of 15 s at 95 °C, and 30 s at 60 °C. In the final step of the protocol, the temperature was raised from 60 to 95 °C at 0.1 °C/step to plot the melting curve.

**Table 2:** Primers used for RT-qPCR

	Cat. no. (Qiagen)	Forward sequence	Reverse sequence
Target gene			
ACTA2	QT00088102	CTATGCCTCTGGACGCACAACT	CAGATCCAGACGCATGATGGCA
COL1A1	QT00037793	GATTCCCTGGACCTAAAGGTGC	AGCCTCTCCATCTTTGCCAGCA
TAGLN	QT00072247	TCCAGGTCTGGCTGAAGAATGG	CTGCTCCATCTGCTTGAAGACC
Housekeeping gene			
GAPDH	QT00079247	GTCTCCTCTGACTTCAACAGCG	ACCACCCTGTTGCTGTAGCCAA

To further analyze the dissociation curves, we visualized the amplicon lengths in an agarose gel to confirm the correct amplification of the resulting PCR products. For the data analysis, the Ct values of each sample and the efficiency of the primer set were calculated through LinReg Software [22] and then converted into relative quantities and normalized using the Pfaffl model [23]. The normalization was performed considering human glyceraldehyde 3-phosphate dehydrogenase (GAPDH) as the housekeeping gene. This gene was chosen according to geNorm software version 3.5 [24].

### Immunofluorescence

Fibroblasts derived from LcSSc/DcSSc patients and from healthy subjects were plated in coverslips in Petri dishes (35 × 10 mm) at a starting low density of 4 × 10^4^ cells/chamber, to prevent possible cell overlapping, and re-suspended in 2 mL of culture medium until 80% of confluence. The cells were processed after 24 h of treatment to evaluate the cytoplasmic localization of α-SMA, Col-I, and SM22. The fibroblasts were washed in PBS (Euroclone) and then fixed in 4% paraformaldehyde solution (Sigma-Aldrich) for 15 min at room temperature. Afterwards, to permeabilize cell membranes, cells were incubated in Triton-X 100 0.2% solution for 30 min at room temperature. Fibroblasts were washed twice in PBS and incubated at 4 °C overnight with anti-human α-SMA (Abcam) diluted at 1:100 in PBS and Triton-X 100 0.05% solution, anti-human type I collagen (Abcam) diluted at 1:100 in PBS and Triton-X 100 0.05% solution and with anti-human SM22 (Abcam) diluted at 1:100 in PBS and Triton-X 100 0.05% solution. Three washes in PBS of the coverslips were followed by 1 h incubation with goat anti-mouse IgG-Texas Red-conjugated antibody (Southern Biotechnology, Italy) diluted at 1:100 in PBS and Triton-X 100 0.05% solution. Cells were then washed twice in PBS and incubated for 10 min with DAPI solution (diluted 1:10000) (Abcam). Finally, the coverslips were mounted with Vecta shield (Vector Labs). Fluorescence was examined under an AxioPlan (Zeiss, Oberkochen, Germany) light microscope equipped with epifluorescence at × 200 and × 400 magnification. The negative controls were obtained by omitting the primary antibody. Immunoreactivity of α-SMA, Col-1, and SM22 were semi-quantified as the mean densitometric area of α-SMA and Col-I signal into the cytoplasm, by AxioVision 4.6 software measure program [25]. At least 100 fibroblasts from each group were evaluated.

### Statistical analysis

Three independent experiments were carried out, and the results were expressed as the mean ± SD of triplicate values for each experiment. Data normal distribution was evaluated by Shapiro–Wilk, D’Agostino and Pearson, and Kolmogorov–Smirnov tests. Data from real-time PCR were evaluated by one-way ANOVA with a Tukey’s post hoc test using 2^−∆∆CT^ values for each sample [26]. All analyses were performed through the SAS System (SAS Institute Inc., Cary, NC, USA) and GraphPad Prism 6.1. A significant value was defined with a *p* value < 0.05.

## Results

### Cell viability and apoptosis

**Fig. 1:**
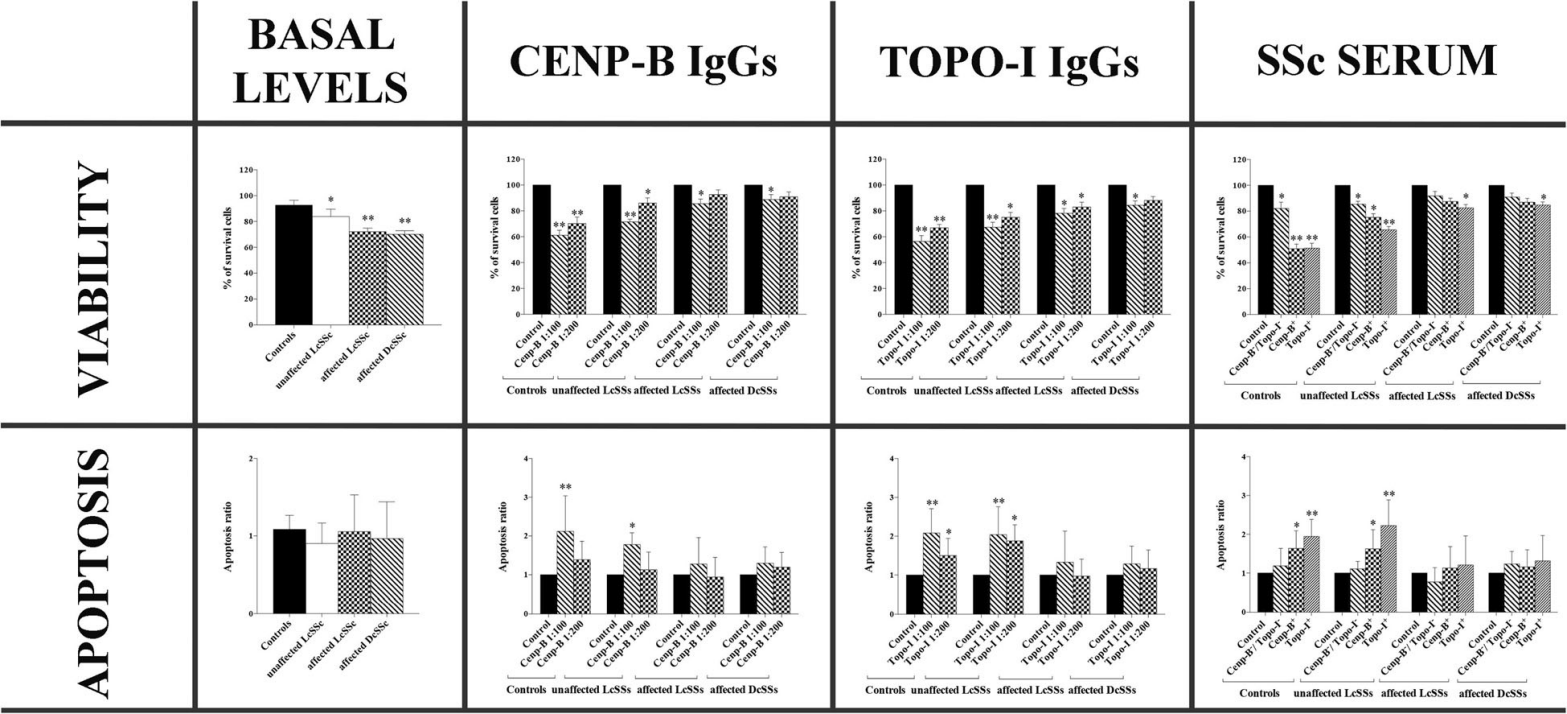
Viability (top row) and apoptosis (bottom row) detected in control, unaffected LcSSc, affected LcSSc, and affected DcSSc fibroblasts at basal levels (untreated) and after stimulation with anti-Cenp-B (ratios 1:100 and 1:200)/anti-Topo-I (ratios 1:100 and 1:200) antibodies and with SSc sera (10% v/v in DMEM). Data were normalized per 10^4^ cells. The statistics is reported with respect to the “Control.” “Control” for IgG stimulations is referred to human healthy control IgGs in a ratio 1:100 in culture medium (DMEM). “Control” for serum stimulation is referred to as human healthy control serum at 10% *v*/*v* in DMEM. (*p < 0.05, **p < 0.01). For SSc serum treatments, Cenp-B-/Topo-I treatment refers to SSc serum negative for all ENAs

Results for viability and apoptosis are reported in Fig. 1. At basal levels, the viability of affected LcSSc and DcSSc fibroblasts resulted decreased compared to control ones. LcSSc-unaffected fibroblasts resulted also a bit less viable than control ones, but not at the levels of LcSSc/DcSSc-affected ones. Anti-Cenp-B and with more extent anti-Topo-I IgGs reduced mainly unaffected LcSSc and control fibroblast (and with less extent affected LcSSc/DcSSc ones) viability in a dilution-dependent manner compared to control IgGs. Similar results were obtained with anti-Cenp B^+^ and anti-Topo-I^+^ sera compared to control sera and to SSc sera negative for anti-Cenp-B, anti-Topo-I antibodies and for other ENAs. Flow cytometry analysis revealed that both anti-Cenp-B/anti-Topo-I IgGs and anti-Cenp B^+^/anti-Topo-I^+^ sera induce apoptosis in unaffected LcSSc and control fibroblasts only, while affected LcSSc and DcSSc fibroblasts showed apoptosis resistance. Anti-histone H3 antibody treatment did not influence viability and apoptosis (Additional file 1). Regarding the IAP, AZD 5582 dihydrochloride showed its efficacy in inhibiting apoptosis and increase viability in control fibroblasts upon stimulation with anti-Cenp-B, anti-Topo-I, and anti-Histone H3 IgGs (Additional file 1).

### Gene expression

**Fig. 2:**
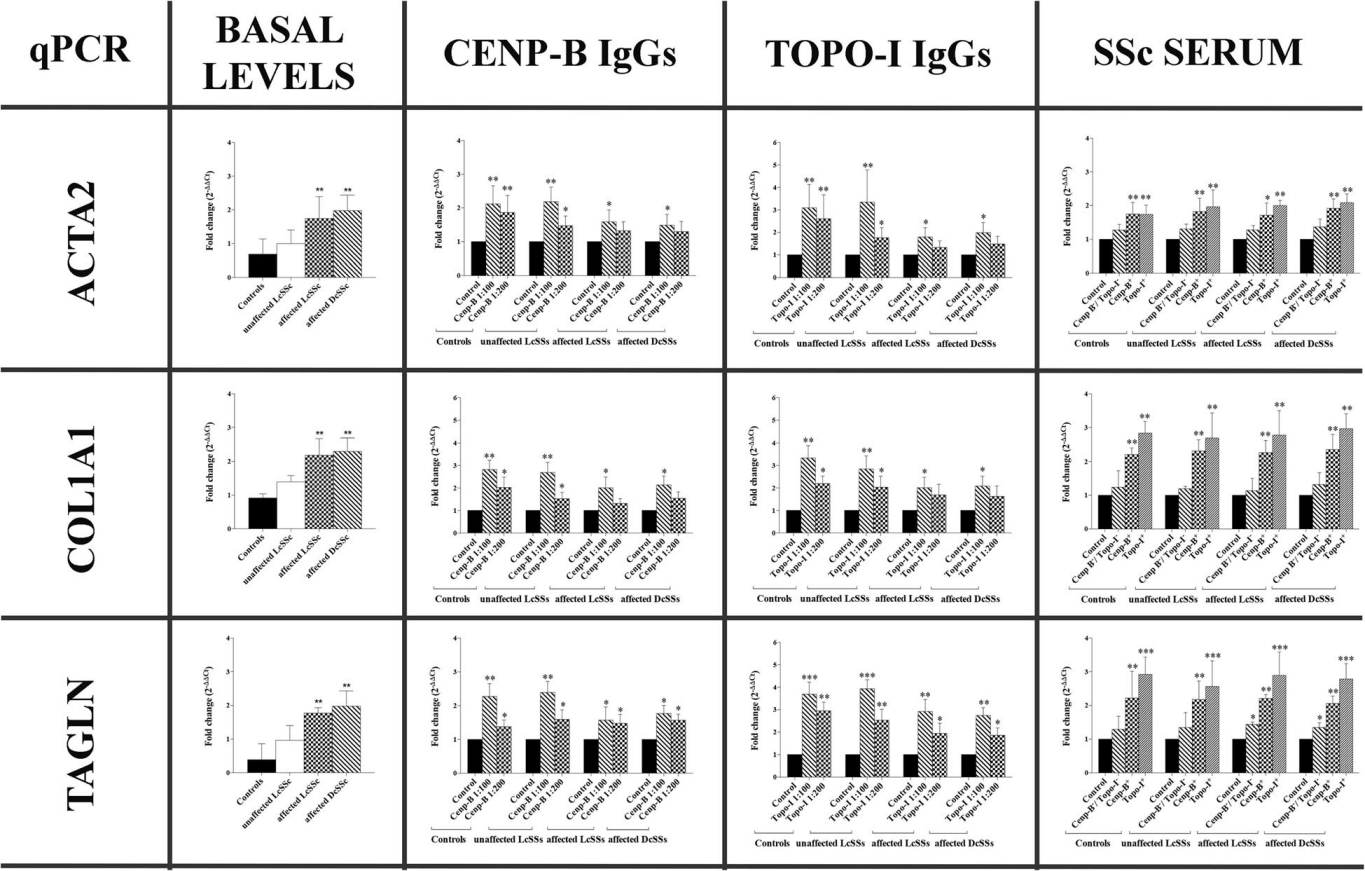
qPCR results for ACTA2 (top row), COL1A1 (middle row), and TAGLN (bottom row) in control, unaffected LcSSc, affected LcSSc, and affected DcSSc fibroblasts at basal levels (untreated) and after stimulation with anti-Cenp-B (ratios 1:100 and 1:200)/anti-Topo-I (ratios 1:100 and 1:200) antibodies and with SSc sera (10% v/v in DMEM). The statistics is reported with respect to the “Control.” “Control” for IgG stimulations is referred to human healthy control IgGs in a ratio 1:100 in culture medium (DMEM). “Control” for serum stimulation is referred to as human healthy control serum at 10% v/v in DMEM. Data are reported as fold change vs “Control” (*p < 0.05, **p < 0.01, ***p < 0.001)

Gene expression levels are reported in Fig. 2. At basal levels, ACTA2, COL1A1, and TAGLN are statistically higher in affected LcSSc and DcSSc fibroblasts compared to control ones. Stimulation with anti-Cenp-B and anti-Topo-I IgGs statistically increased all the profibrotic markers compared to control IgGs. Control and unaffected LcSSc fibroblasts seem to be more prone to IgG stimulation than affected LcSSc/DcSSc ones. Stimulation with anti-Cenp-B^+^ and anti-Topo-I^+^ sera increased ACTA2, COL1A1, and TAGLN expressions compared to control sera and to SSc Cenp-B^−^/Topo-I^−^ sera for all the fibroblast populations. Finally, inhibition of apoptosis did not change the mRNA upregulation of all the pro-fibrotic markers upon stimulation with anti-Cenp-B, anti-Topo-I, and anti-Histone H3 IgGs in control fibroblasts (Additional file 1).

### Immunofluorescence

**Fig. 3:**
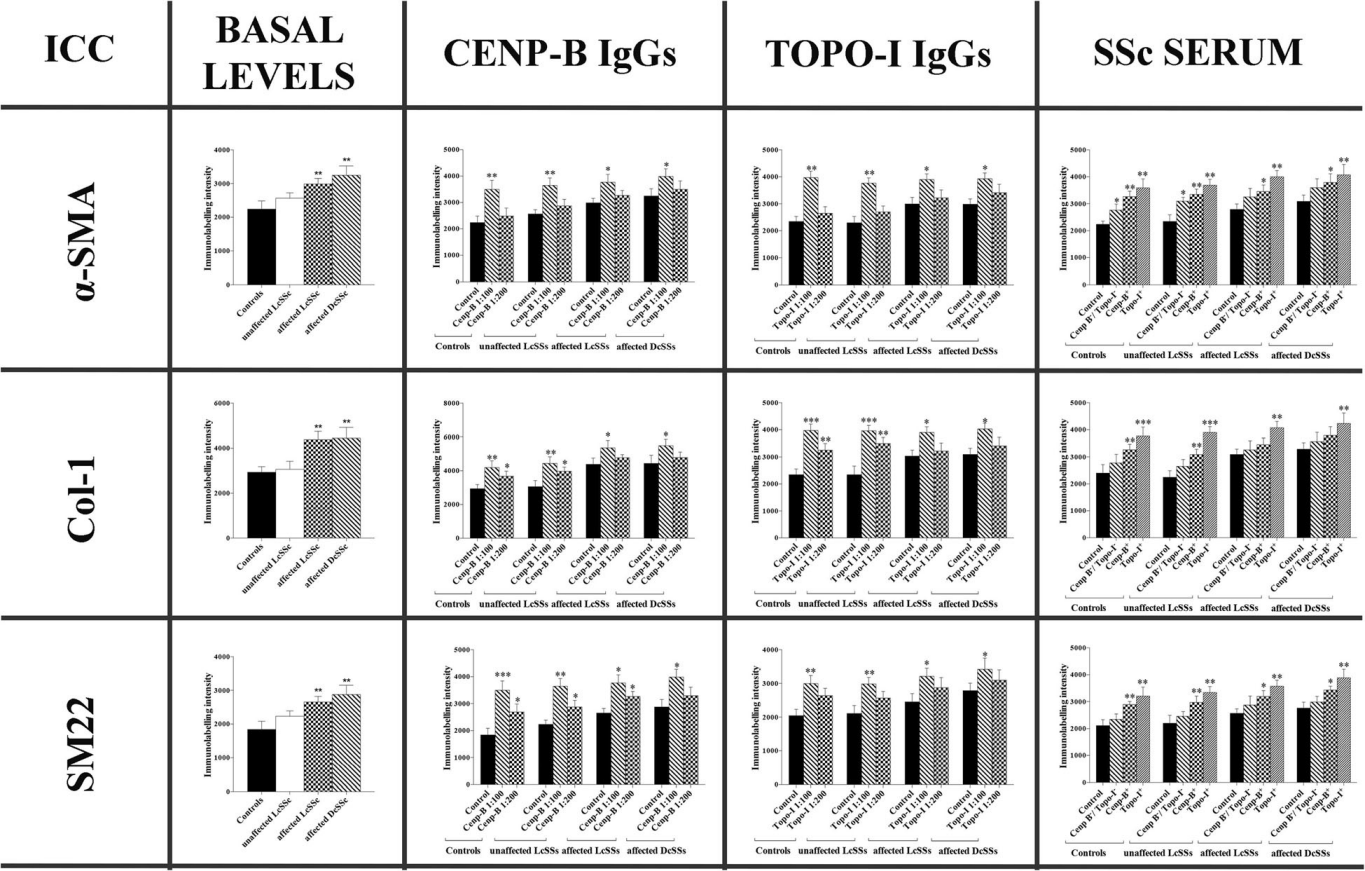
ICC results for α-SMA (top row), Col-1 (middle row), and SM22 (bottom row) in control, unaffected LcSSc, affected LcSSc and affected DcSSc fibroblasts at basal levels (untreated) and after stimulation with anti-Cenp-B (ratios 1:100 and 1:200)/anti-Topo-I (ratios 1:100 and 1:200) antibodies and with SSc sera (10% *v*/*v* in DMEM). The statistics is reported with respect to the “Control.” “Control” for IgG stimulations is referred to human healthy control IgGs in a ratio 1:100 in culture medium (DMEM). “Control” for serum stimulation is referred to as human healthy control serum at 10% *v*/*v* in DMEM. Data are reported as Immunolabeling Intensity vs “Control”. Immunolabeling Intensity corresponds to the formula *I* × *A*/*n* where *I* = intensity levels, *A* = area, *n* = number of cells (*p < 0.05, **p < 0.01, ***p < 0.001)

**Fig. 4:**
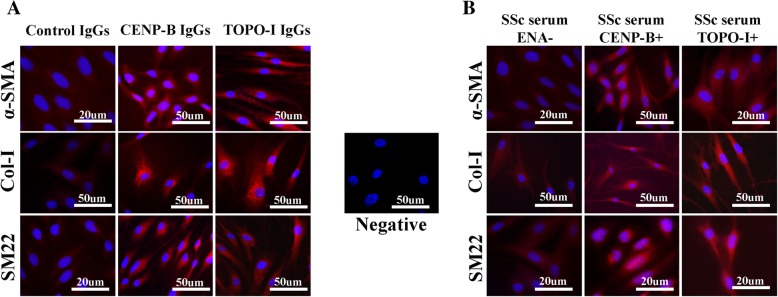
ICC representative images for all the three profibrotic markers α-SMA (top row), Col-1 (middle row), and SM22 (bottom row) in control fibroblasts stimulated with IgGs (**a**) and with sera (**b**). For IgG stimulation (**a**), data are represented for ratio 1:100 only due to the better window shown in qPCR and ICC quantification. For serum stimulation (**b**), data are represented with respect to SSc sera negative for anti-Cenp-B and anti-Topo-I antibodies and for other ENAs. Negative control is represented in the middle and obtained by replacing the primary antibody with PBS

Results for immunofluorescence are shown in Fig. 3. At basal levels (before the treatment), α-SMA, Col-I, and SM22 are statistically higher in affected LcSSc and DcSSc fibroblasts compared to control ones. Stimulation with anti-Cenp-B and anti-Topo-I IgGs and with anti-Cenp-B^+^ and anti-Topo-I^+^ sera statistically increased all the profibrotic markers compared to control IgGs, to control sera and to SSc sera negative for anti-Cenp-B and anti-Topo-I antibodies and for other ENAs. In Fig. 4, representative images of ICC assay for all the three markers (α-SMA, Col-I, and SM22) confirm consistency with the ICC quantification data. Same as for qPCR, inhibition of apoptosis did not change the protein upregulation of all the pro-fibrotic markers upon stimulation with anti-Cenp-B, anti-Topo-I, and anti-Histone H3 IgGs in control fibroblasts (Additional file 1).

## Discussion

To the best of our knowledge, this is the first study in which the direct effect of antibodies targeting SSc-specific ENAs that are anti-Cenp-B and anti-Topo-I has been evaluated on the pro-fibrotic activation of cultured human dermal fibroblasts and their subsequent differentiation into a myofibroblast phenotype in vitro. SSc-specific antibodies have been used mainly as indicators of clinical subsets of the disease [27]. Moreover, these antibodies are as important tools for the prediction of possible organ involvement [28]. However, very little is known about their direct pathogenic effect on different cell phenotypes in the disease [29]. What is known in the population of SSc antibody patients is that patients with anti-Cenp-B antibodies (usually LcSSc patients) more frequently develop pulmonary arterial hypertension (PAH) and prolonged gastrointestinal transit time [30], while SSc patients with anti-Topo-I antibodies (usually DcSSc patients) are linked with a higher probability of interstitial lung disease (ILD), renal vascular damage, renal crisis, and heart fibrosis [31]. All these internal organ complications involve the fibroblast as the key effector cell phenotype driving the fibrotic process in SSc [32]: therefore, there must be a direct and/or indirect link between the presence of anti-Cenp-B/anti-Topo-I antibodies and the pro-fibrotic activation of fibroblasts. In literature, there are some hypotheses on how those antibodies could indirectly mediate the fibrotic development in SSc [33, 34]. Among these, the hypothesis that SSc-specific antibodies could trigger the fibrotic development by inducing microvascular alterations and subsequent tissue remodeling is one of the most reliable [35]. Another important hypothesis is that SSc-specific antibodies form immune complexes (ICs) upon their interaction with soluble target antigens [36]: it has been demonstrated that ICs containing anti-Cenp-B/anti-Topo-I antibodies induce a pro-fibrotic and pro-inflammatory phenotype in dermal fibroblasts [37]. Particularly, scientists demonstrated that Topo-I binding to fibroblast surfaces is both necessary and sufficient for anti-Topo-I binding [38]. Second, Topo-I/anti-Topo-I complex binding can then trigger the adhesion and activation of monocytes, thus providing a plausible model for the amplification of the fibrogenic cascade in anti-Topo-I-positive SSc patients [39]. To some extent, this model looks very artificial since Topo-I should be an intracellular antigen (it is usually located in the nucleus) and not an extracellular one [40]. However, subsequent in vivo studies demonstrated that Topo-I released from injured endothelial cells could bind to bystander fibroblasts thus displaying chemoattractant activity toward immature dendritic cells and human monocytes [41]. These results represent a clear demonstration that Topo-I could display an extracellular role that can affect fibroblast physiology. Similar results were found for Cenp-B antigen [42]: in particular, scientists demonstrated that Cenp-B released from apoptotic endothelial cells in vivo binds more specifically to the surface of human pulmonary artery smooth muscle cells (SMCs) than fibroblasts [43]; in particular, Cenp-B binds preferentially to SMCs of the contractile type rather than to SMCs of the synthetic type [44]. The different Cenp-B selectivity of binding to SMCs rather than to fibroblasts could explain our results showing that anti-Topo-I antibodies have a stronger effect on fibroblasts than anti-Cenp-B ones. The different target cells of Cenp-B (SMCs) and Topo-I (fibroblasts) autoantigens released by apoptotic endothelial cells could also partially explain the different complications and organ involvement between LcSSc and DcSSc [45]. Both Cenp-B and Topo-I models assume an already established endothelial damage as a source of autoantigens binding to targeting cells and generating a specific pathogenic autoantibody (anti-Cenp-B and anti-Topo-I) response. However, these models are quite in disagreement with the timing of the disease evolution since those specific circulating autoantibodies could be detected in SSc patients before an established endothelial damage [46]. This is also the reason why in the past the use of Topo-I inhibitors for the treatment of SSc patients resulted unsuccessful [47]. With the results of the present study, we assume that anti-Cenp-B and anti-Topo-I antibodies could exert a direct pathogenic role, and therefore, they could be considered “functional antibodies.” In fact, an autoantibody is considered “functional” if its direct interaction with an identified target antigen leads to a molecular pathway activation or inhibition that can be replicated in an experimental setting [48]. Therefore, the results of the present study suggest a new interpretation of the role of anti-Cenp-B and anti-Topo-I antibodies in the disease in terms of disease drivers and not only representing an epiphenomenon and/or useful diagnostic and prognostic tools. In fact, we believe that these main autoantibodies, when present in the circulation even years before the clinical involvement, need to be tackled to slow down or prevent disease development. To prove this hypothesis, we have support from literature: it has been demonstrated that ubiquitous nuclear protein Cenp-B is the main target of anti-endothelial cell antibodies (AECA) in patients with LcSSc and that AECA from DcSSc patients bind to endothelial cell topoisomerase I, suggesting that classical autoantibodies such as anti-Cenp-B and anti-Topo-I antibodies could act as AECA inducing cell-mediated toxicity and apoptosis in the early stages of the disease [49]. In this scenario, the use of immunosuppressive drugs and/or the development of more specific drugs targeting anti-Cenp-B and anti-Topo-I antibodies should be recommended at an early stage of the disease to prevent future organ damage or decrease the fibrotic evolution. In this regard, we underline that in the literature, to our knowledge, there are no studies on the progress and the possible decrease of the autoantibody titer during immunosuppressive therapy. Another important finding evidenced in this work is the different responsiveness of the fibroblasts to those autoantibodies according to their differentiation stage: healthy and unaffected LcSSc fibroblasts were more prone to be activated upon stimulation with anti-Cenp-B and anti-Topo-I antibodies than affected LcSSc and DcSSc fibroblasts that resulted already differentiated into activated myofibroblasts, thus secreting the maximum level of pro-fibrotic proteins. On the other hand, healthy and unaffected LcSSc fibroblasts were also more susceptible to apoptosis than affected LcSSc and DcSSc ones, suggesting that the fate of fibroblasts depends not only on autoantibodies but on a combination of specific autoantibodies (e.g., anti-histone antibodies did not induce apoptosis) and other soluble factors: in fact what we call healthy or unaffected LcSSc fibroblasts are cells that, upon stimulation with autoantibodies, they secrete a maximum amount of pro-fibrotic proteins and then undergo apoptosis. On the other hand, what we call affected LcSSc and DcSSc fibroblasts are cells that already express high amount of pro-fibrotic proteins and, upon stimulation with autoantibodies, they keep producing collagen and other contractile proteins but with apoptosis resistance, so the self-sustained pro-fibrotic loop is established [50]. This theory is in line with recently published literature in which it has been demonstrated that mitochondria in activated myofibroblasts, but not quiescent fibroblasts, are primed by death signals (proximity to the apoptotic threshold) which creates a requirement for tonic expression of the antiapoptotic proteins to ensure myofibroblast survival [51]. In this irreversible loop, the inefficient removal of nuclear components of cells targeted by the autoantibodies (defective cellular “waste disposal” theory) may also lead to the release and prolonged exposure of nuclear components and thus to the generation of new autoantibodies with increased or stable titers in sera of SSc patients.

## Conclusions

In conclusion, we suggest to re-consider the use of SSc-specific antibodies not only as useful diagnostic and prognostic tools, but also as therapeutic targets of the disease itself. We acknowledge that our study presents intrinsic limitations. Being an in vitro study, it might be oversimplified, not allowing an adequate reproduction of the complexity of the disease pathogenesis. Moreover, the direct pathogenic role of other SSc-related autoantibodies (e.g., anti-RNA polymerase III) needs to be further investigated.

## Additional file

Additional file 1: Viability and apoptosis (left panel) and qPCR and ICC (right panel) data regarding control fibroblasts stimulated with specific SSc-autoantibodies (anti-Cenp-B, anti-Topo-I IgGs 1:100) and with SSc-unrelated one (anti-Histone H3 IgGs 1:100) with and without the pre-incubation (2 h) with an anti-apoptotic compound (IAP, AZD 5582 dihydrochloride, 50 nM) (*p < 0.05). (TIF 42160 kb)

## Data Availability

Please contact the author for data requests.

## Abbreviations

ACA: Anti-centromere antibody
ACR: American College of Rheumatology
AECA: Anti-endothelial cell antibodies
ANA: Anti-nuclear antibody
Anti-Cenp-B: Anti-centromeric protein B
Anti-eIF2B: Anti-eukaryotic initiation factor 2B
Anti-RNAP: Anti-RNA polymerase III
Anti-RNP: Anti-U3 ribonucleoprotein
Anti-Topo-I: Anti-DNA-topoisomerase-I
Col-I: Type I collagen
DcSSc: Diffuse cutaneous systemic sclerosis
DMEM: Dulbecco’s modified Eagle’s medium
DMSO: Dimethyl sulfoxide
ENA: Extractable nuclear antigen
EULAR: European league against rheumatism
FBS: Fetal bovine serum
FITC: Fluorescein isothiocyanate
GAPDH: Glyceraldehyde 3-phosphate dehydrogenase
IC: Immune complex
ICC: Immunocytochemistry
ILD: Interstitial lung disease
LcSSc: Limited cutaneous systemic sclerosis
MTT: 3-(4,5-Dimethylthiazol-2-yl)-2,5-diphenyltetrazolium bromide
OD: Optical density
PAH: Pulmonary arterial hypertension
PBS: Phosphate-buffered saline
PI: Propidium iodide
qPCR: Quantitative real-time polymerase chain reaction
SD: Standard deviation
SM22: Trangelin
SMC: Smooth muscle cell
SSc: Systemic sclerosis
α-SMA: Alpha smooth muscle actin
