# Supplementary figures and images for: Antibodies against specific extractable nuclear antigens (ENAs) as diagnostic and prognostic tools and inducers of a profibrotic phenotype in cultured human skin fibroblasts: are they functional?
Source: Arthritis Res Ther. 2019 Jun 24;21:152. doi: 10.1186/s13075-019-1931-x (PMC6592008; doi:10.1186/s13075-019-1931-x)

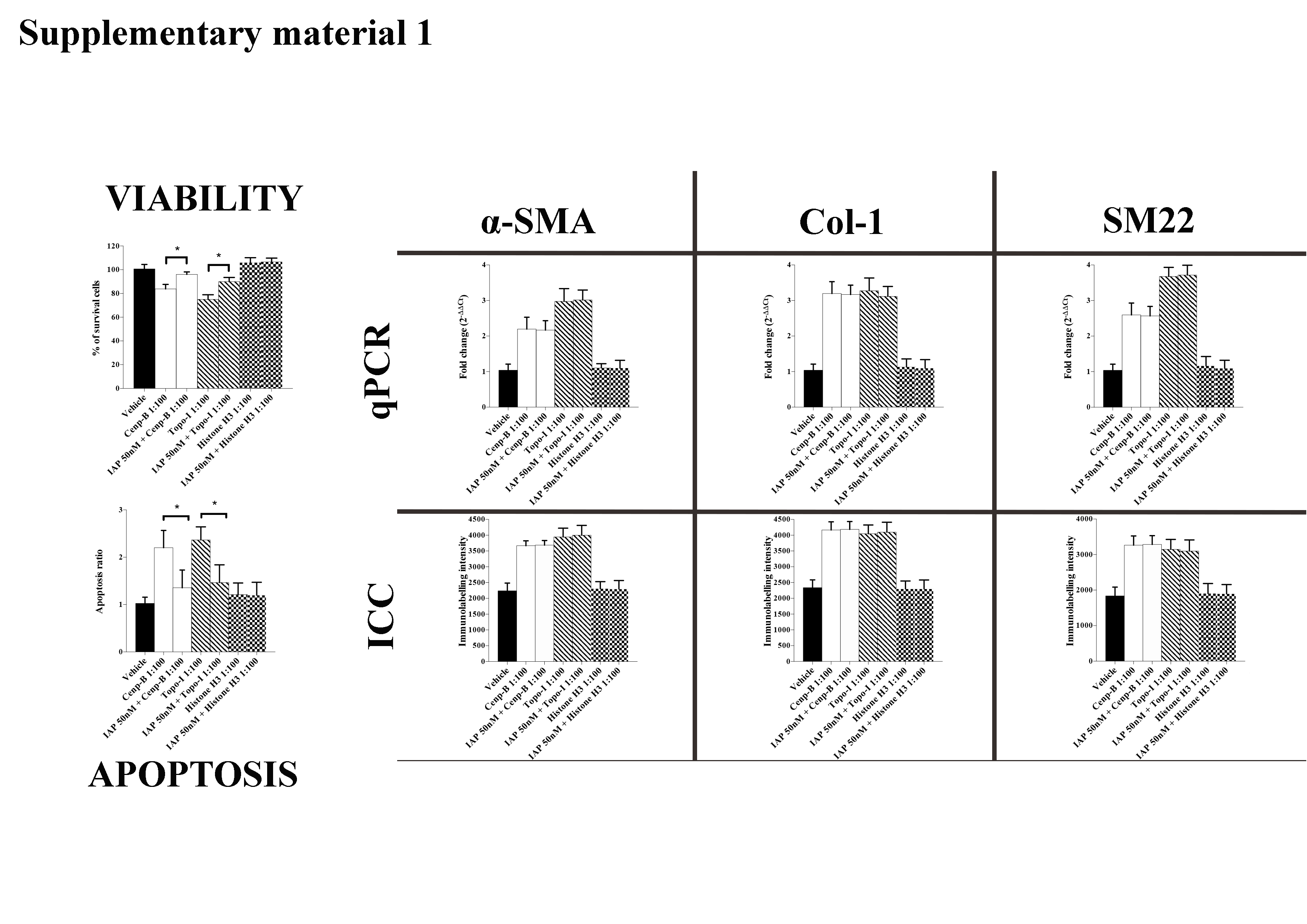

Supplement: Supplementary file 1 — Viability and apoptosis (left panel) and qPCR and ICC (right panel) data regarding control fibroblasts stimulated with specific SSc-autoantibodies (anti-Cenp-B, anti-Topo-I IgGs 1:100) and with SSc-unrelated one (anti-Histone H3 IgGs 1:100) with and without the pre-incubation (2 h) with an anti-apoptotic compound (IAP, AZD 5582 dihydrochloride, 50 nM) (*p < 0.05). (TIF 42160 kb) [file 13075_2019_1931_MOESM1_ESM.tif]
